# The Differentiation Stage of Transplanted Stem Cells Modulates Nerve Regeneration

**DOI:** 10.1038/s41598-017-17043-4

**Published:** 2017-12-12

**Authors:** Ching-Wen Huang, Wen-Chin Huang, Xuefeng Qiu, Flavia Fernandes Ferreira da Silva, Aijun Wang, Shyam Patel, Leon J. Nesti, Mu-Ming Poo, Song Li

**Affiliations:** 1 Department of Bioengineering, University of California, Berkeley, California, 94720 USA; 2 UC Berkeley-UCSF Graduate Program in Bioengineering, Berkeley, California, 94720 USA; 3 Department of Cardiovascular Surgery, Union Hospital, Tongji Medical College, Huazhong University of Science and Technology, Wuhan, 430022 China; 4 Instituto de Macromoléculas Professora Eloisa Mano, Universidade Federal do Rio de Janeiro, Rio de Janeiro, Brazil; 5 Department of Surgery, University of California, Davis, School of Medicine, Sacramento, California, 95817 USA; 6 Department of Surgery, Uniformed Services University of the Health Sciences, Bethesda, Maryland 20814 USA; 7 Clinical and Experimental Orthopaedics, National Institute of Arthritis and Musculoskeletal and Skin Diseases, National Institutes of Health, Bethesda, Maryland 20892 USA; 8 Department of Orthopaedic Surgery, Walter Reed National Military Medical Center, Bethesda, Maryland 20889 USA; 9 Department of Molecular and Cell Biology, University of California, Berkeley, California, 94720 USA; 10 Department of Bioengineering, University of California, Los Angeles, California, 90095 USA; 11 Department of Medicine, University of California, Los Angeles, California, 90095 USA

## Abstract

In regenerative medicine applications, the differentiation stage of implanted stem cells must be optimized to control cell fate and enhance therapeutic efficacy. We investigated the therapeutic potential of human induced pluripotent stem cell (iPSC)-derived cells at two differentiation stages on peripheral nerve regeneration. Neural crest stem cells (NCSCs) and Schwann cells (NCSC-SCs) derived from iPSCs were used to construct a tissue-engineered nerve conduit that was applied to bridge injured nerves in a rat sciatic nerve transection model. Upon nerve conduit implantation, the NCSC group showed significantly higher electrophysiological recovery at 1 month as well as better gastrocnemius muscle recovery at 5 months than the acellular group, but the NCSC-SC group didn’t. Both transplanted NCSCs and NCSC-SCs interacted with newly-growing host axons, while NCSCs showed better survival rate and distribution. The transplanted NCSCs mainly differentiated into Schwann cells with no teratoma formation, and they secreted higher concentrations of brain-derived neurotrophic factor and nerve growth factor than NCSC-SCs. In conclusion, transplantation of iPSC-NCSCs accelerated functional nerve recovery with the involvement of stem cell differentiation and paracrine signaling. This study unravels the *in vivo* performance of stem cells during tissue regeneration, and provides a rationale of using appropriate stem cells for regenerative medicine.

## Introduction

Induced pluripotent stem cells (iPSCs) are derived from somatic cells that have been reprogrammed back into an embryonic-like pluripotent state. The generation of iPSCs^1–7^, especially iPSCs without the integration of reprogramming factors into the genome^8–16^, makes it possible for patient-specific cell therapies, which may bypass immune rejection issues and ethical concerns for the usage of embryonic stem cells (ESCs). For therapeutic use in tissue regenerative applications, the specific differentiation state of implanted iPSCs must be optimized to control cell fate, viability, potency and safety *in vivo*. Therefore, it is important to use appropriate differentiation stage of the cells for a specific therapy, as well as understanding the mechanism of stem cell differentiation and the functional activities during the regeneration of specific tissue.

To address these critical issues of stem cell therapies, in this study, we specifically investigated the impact of different stages of iPSC-derived neural lineage cells on peripheral nerve regeneration in a rat sciatic nerve transection model. Peripheral nerve injuries following traumatic injuries and tumor removal surgeries often requires surgical repair. Disadvantages of using nerve autograft^17,18^ include morbidity at the donor site and the unavailability of autograft. Synthetic nerve conduits with aligned nanofibers have shown great potential to guide axon growth^19–22^, but it takes 2–3 months for the functional recovery of transected nerves across a 1-cm gap and the regeneration across a gap >3 cm is difficult, which may result in the degeneration and dysfunction of muscle and tissues lack of innervation. There is evidence that Schwann cells can enhance axon growth and myelination^23–26^. However, harvesting adult Schwann cells is difficult and causes morbidity at the donor site. Neural crest stem cells (NCSCs), a source of Schwann cells^27–30^, are multipotent stem cells that can be isolated from ESCs and embryonic neural crest but have relative low abundance in adult tissues^27,31,32^. We and others have shown that NCSCs can be differentiated from iPSCs^33–35^, and further differentiate into cell types of all three germ layers, e.g., neurons, Schwann cells, vascular smooth muscle cells (SMCs), bone cells, cartilage cells, melanocytes and endocrine cells, which makes NCSCs a valuable stem cell source for tissue regeneration and an ideal model system to study the lineage commitment and therapeutic potential of stem cells. Previous studies have shown that transplantation of iPSC-induced NCSCs in nerve conduits promotes nerve regeneration at 1 month^36^, but those iPSCs are not integration-free and the underlying mechanisms are not clear.

In this study, we generated human integration-free iPSCs from human dermal fibroblasts by using electroporation, and then differentiated the iPSCs into NCSCs and finally into Schwann cells (NCSC-SCs). The therapeutic effects of the cells at various differentiation stages were investigated and compared in a rat sciatic nerve transection model wherein the nerve injury was bridged with a poly(L-lactide-co-caprolactone) (PLCL) nanofibrous conduit. The mechanisms of transplanted cells-induced nerve regeneration, including *in vivo* differentiation and paracrine signaling, were further studied.

## Results

### Characterization of Human Integration-free iPSC Lines and iPSC-derived NCSCs and Schwann Cells

**Figure 1:**
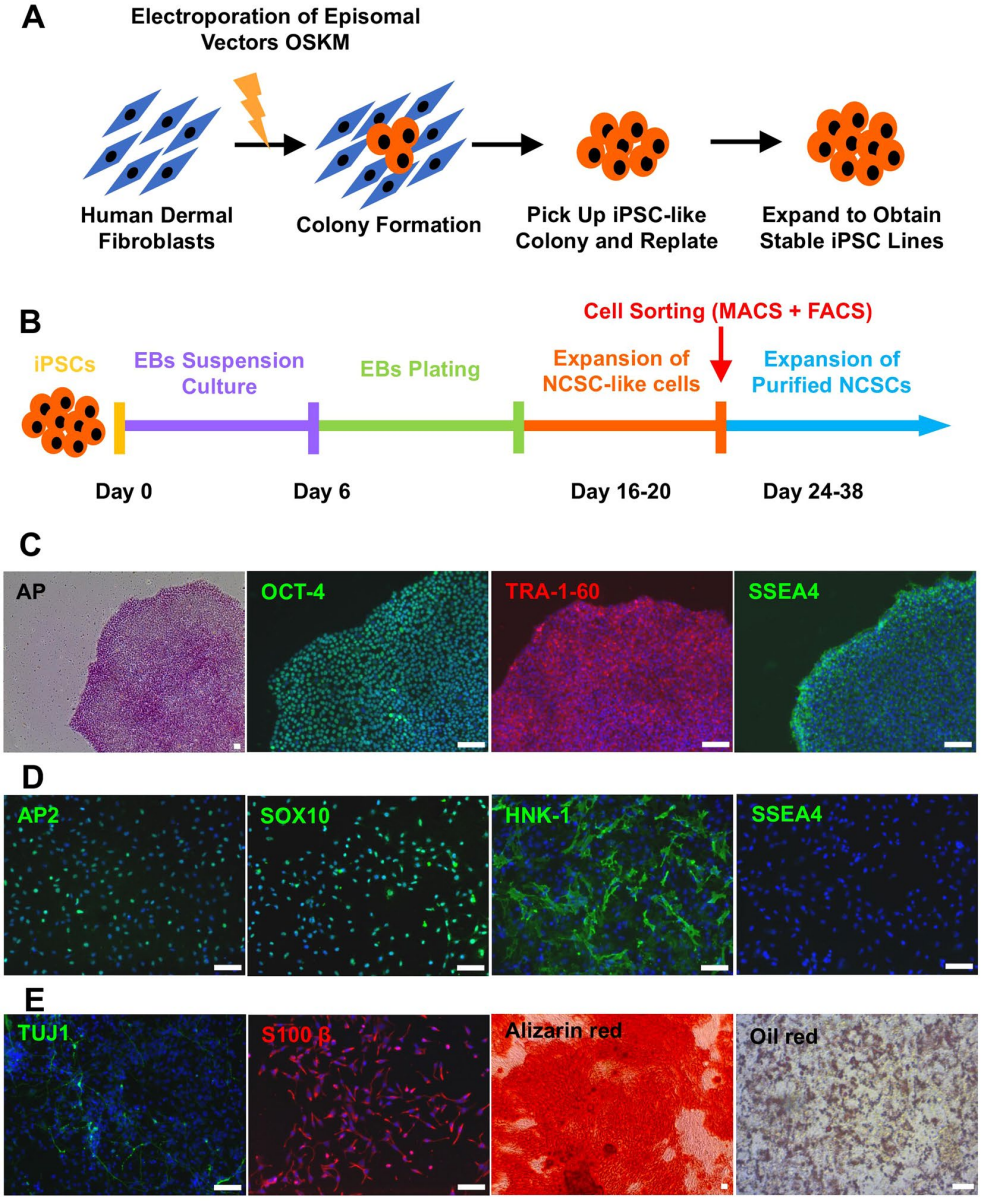
Establishment and characterization of human integration-free iPS cell lines and iPSC-derived NCSCs and Schwann cells. (**A**) Human dermal fibroblasts were reprogrammed with episomal vectors containing Oct4, Sox2, Klf4, and c-Myc genes by using electroporation method. Pluripotent stem cell-like colonies were picked up and expanded to obtain stable iPSC lines. (**B**) To establish NCSC lines, iPSCs were detached and formed embryoid bodies (EBs) in suspension cultures. EBs were then plated on Matrigel-coating culture plates for up to 2 weeks. Subsequently, cells were dissociated into single cells and cultured as monolayer. To obtain homogeneous NCSC populations, magnetic-activated cell sorting (MACS) were used to select p75 positive cells. Expended p75+ NCSCs were further purified by fluorescence-activated cell sorting (FACS) for HNK-1 positive and SSEA4 negative cells to obtain more homogeneous and stable NCSC line. (**C**) Established iPSC lines showed typical pluripotent stem cell morphology, positive AP staining, and iPSC markers OCT-4, SSEA4, and TRA-1-60. (**D**) The iPSC-derived NCSC lines showed positive NCSC markers SOX10, HNK-1, and AP2 and negative iPSC marker SSEA4. (**E**) *In vitro* differentiation of iPSC-derived NCSCs into peripheral neural lineages (peripheral neurons, TUJ1; Schwann cells, S100β) and mesenchymal lineages (osteoblasts, Alizarin red; adipocytes, Oil red). Nuclei were stained by Hoechst 33342. Scale bar: 50 μm.

Human dermal fibroblasts were reprogrammed with Yamanaka factors delivered by electroporation to generate integration-free human iPSCs (Fig. 1A), and the fully characterized iPSC lines were used to derive NCSCs (Fig. 1B) by using an optimized protocol. Established human integration-free iPSC lines showed typical pluripotent stem cell morphology, positive alkaline phosphatase (AP) staining, and positive expression of iPSC markers OCT-4, SSEA4, and TRA-1-60 (Fig. 1C). The iPSC-NCSC lines stained positive for NCSC markers SOX10, HNK-1, and AP2, and negative for the iPSC marker SSEA4 (Fig. 1D). *In vitro* differentiation showed that the iPSC-NCSCs were able to differentiate into peripheral neural lineages and mesenchymal lineages (Fig. 1E). Positive expression of neuron marker TUJ-1 (peripheral nerve differentiation) and Schwann cell marker S100β (Schwann cell differentiation) was observed after 2-week NCSC differentiation in conditioned media. Mesenchymal lineage differentiation was verified by positive Alizarin red staining for calcium precipitation and positive oil red lipid staining in NCSC-derived osteoblast and adipocyte cultures, respectively, following a 3-week differentiation protocol.

**Figure 2:**
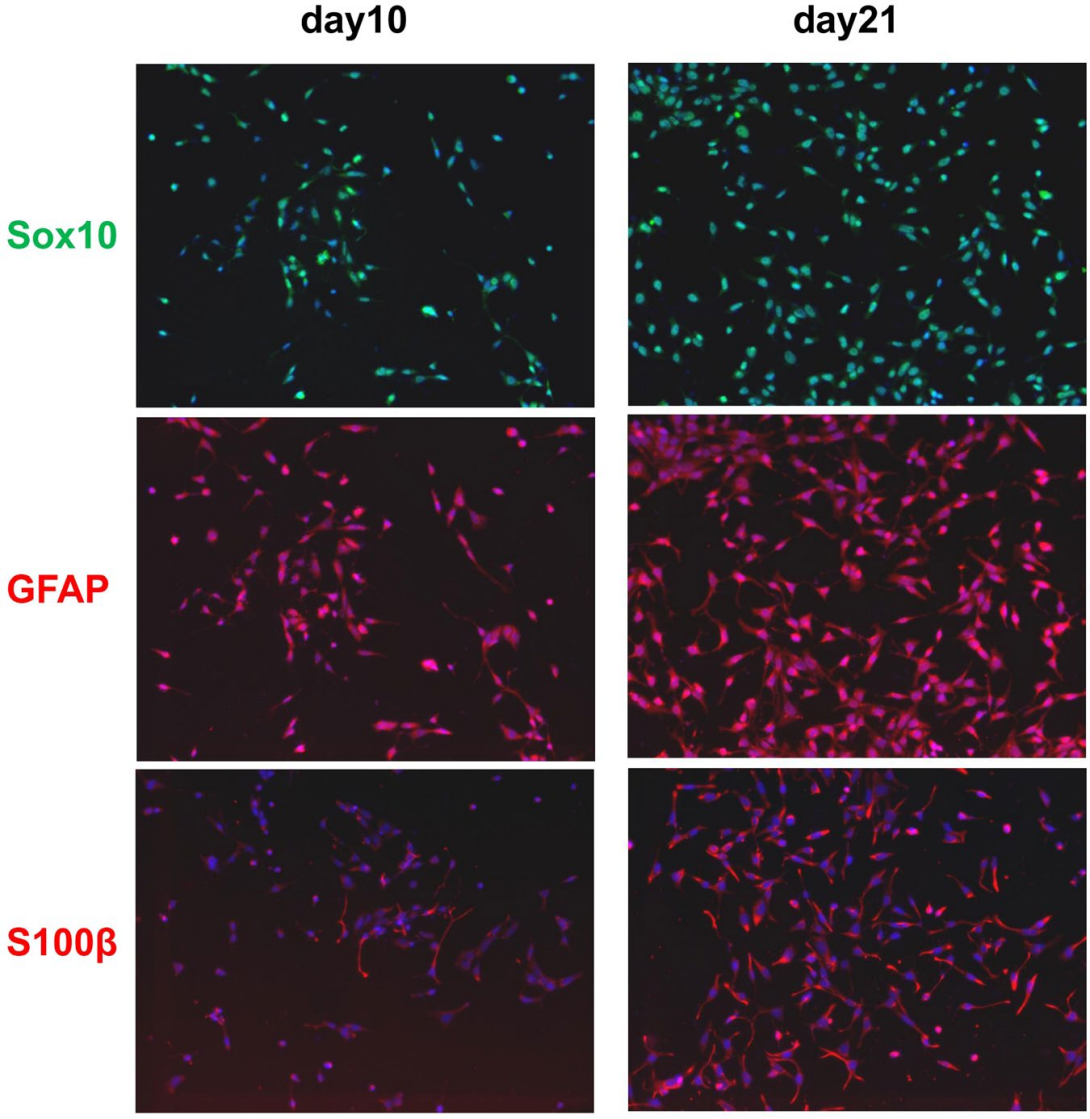
Marker expression of 10-day and 21-day differentiated iPSC-NCSCs in Schwann cell differentiation medium.

To obtain Schwann cells from NCSCs (NCSC-SCs), we compared the expression of Schwann cell markers at day 10 and day 21 of NCSC-SC differentiation (Fig. 2). At day 21, majority of the cells showed positive S100β and GFAP staining. We then used NCSC-SCs at day 21 for the *in vivo* studies to compare the therapeutic effects with undifferentiated NCSCs.

### *In Vivo* Evaluation of Nerve Functional Recovery

**Figure 3:**
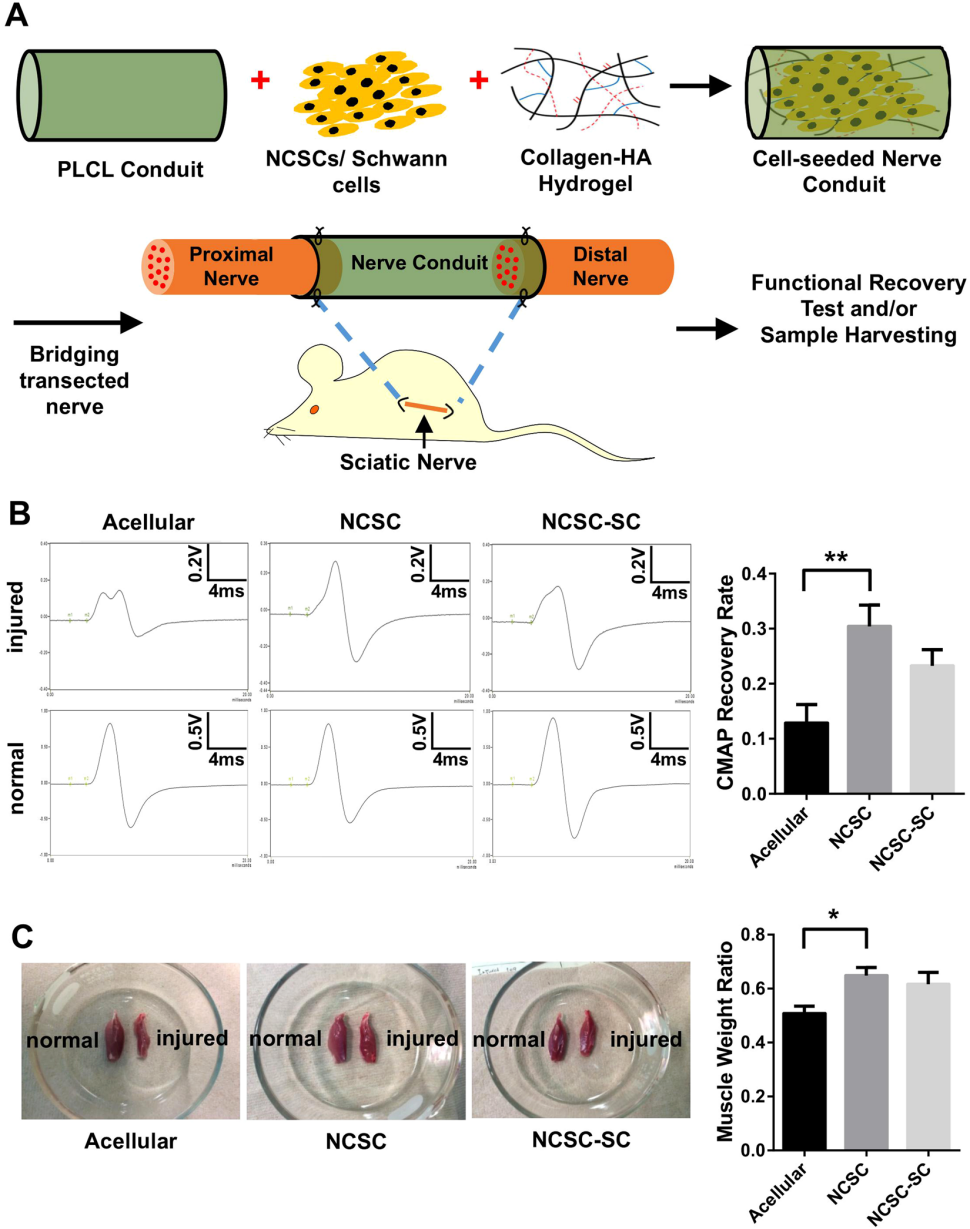
Transplantation of NCSCs and NCSC-SCs for peripheral nerve regeneration and *in vivo* evaluation of functional recovery. (**A**) Schematic outline of tissue engineering approach by combining NCSCs/NCSC-SCs, collagen/HA hydrogel, and a PLCL nerve conduit. The NCSCs/NCSC-SCs were mixed with collagen-hyaluronic acid (Col-HA) hydrogel, injected in the nerve conduits, and incubate overnight *in vitro*. The nerve conduits were then used to connect the cut sciatic nerves in nude rats. (**B**) Compound muscle action potential (CMAP) was measured *in vivo* at 1-month after surgery. Representative CMAP curves of the acellular, NCSC, and NCSC-SC groups are shown. Recovery rate is the ratio of injured hindlimb’s CMAP to contralateral normal hindlimb’s CMAP of a rat. Bars represent mean ± standard error of mean. ** indicates significant difference (p < 0.01; n = 6) (**C**) Five months after surgery, gastrocnemius muscle wet weight was measured and compared between injured hindlimb and contralateral normal hindlimb of a rat. Representative images of gastrocnemius muscle are shown for the acellular group, the NCSC group, and the NCSC-SC group. Bars represent mean ± standard error of mean. * indicates significant difference (p < 0.05; n = 5 for acellular group and NCSC group, and n = 6 for NCSC-SC group).

Nerve conduits containing human iPSC-derived NCSCs or NCSC-SCs, polymer tube, and hydrogel matrix were prepared in tissue culture hood and then transplanted into nude rats to connect the cut sciatic nerves in the right hindlimbs (Fig. 3A). To assess nerve functional recovery following graft implantation, electrophysiology testing was performed *in vivo* one month after surgery. Compound muscle action potentials (CMAPs) of the injured sciatic nerve and the contralateral intact nerve were measured and compared (Fig. 3B). After 1-month recovery, CMAPs were detected in 83% of the animals in all groups (5 out of 6 rats for each group). For the rats with detectable CMAPs, the recovery rate of each rat was calculated as a percentage of the measured values from the normal contralateral sciatic nerve. The mean recovery rate of NCSC-engrafted group, NCSC-SC-engrafted group, and acellular group at 1 month were 30.4 ± 3.9%, 23.3 ± 2.9%, and 12.9 ± 3.4%, respectively. Significant difference was observed between NCSC group and acellular group (p < 0.01) but not between NCSC-SC group and acellular group.

Motor nerve conduction testing was used as an indicator to assess nerve recovery, which was performed by measuring the response latency of injured sciatic nerve and contralateral non-injured sciatic nerve. The latency recovery rate was determined by a percentage of the response time of injured nerve to that of contralateral non-injured nerve. The results showed that the latency recovery rate of acellular group, NCSC group, and NCSC-SC group at 1 month were all over 75% with no significant difference (Figure S1).

At five months after surgery, axons already grew across nerve conduits, but the neuromuscular function recovery as determined by gastrocnemius muscle wet weight measurement still showed significant difference. Muscle mass outcomes were reported as proportions between operated/contralateral sides (Fig. 3C). The mean muscle weight ratio in NCSC-engrafted group was significantly higher than the acellular group (p < 0.01), implying that the accelerated recovery of nerve function might help maintaining muscle mass. No teratoma formation was observed at all time points.

To determine whether other stem cell type could promote nerve regeneration, we transplanted human traumatized muscle-derived mesenchymal stem cells (M-MSCs) into the nerve conduits because M-MSCs had been shown to secrete neurotrophic factors such as brain-derived neurotrophic factor (BDNF) and ciliary neurotrophic factor (CNTF) *in vitro* ^37,38^. Our results revealed that the M-MSC group did not have better recovery than the acellular group at 1-month after surgery (Figure S2). In addition, there was no significant difference in muscle weight ratio between the M-MSC and the acellular group (Figure S3), suggesting that other neurotrophic factors and/or other stem cell functions might be needed to promote nerve regeneration.

### Cell Fate of Transplanted cells in Nerve Conduit

**Figure 4:**
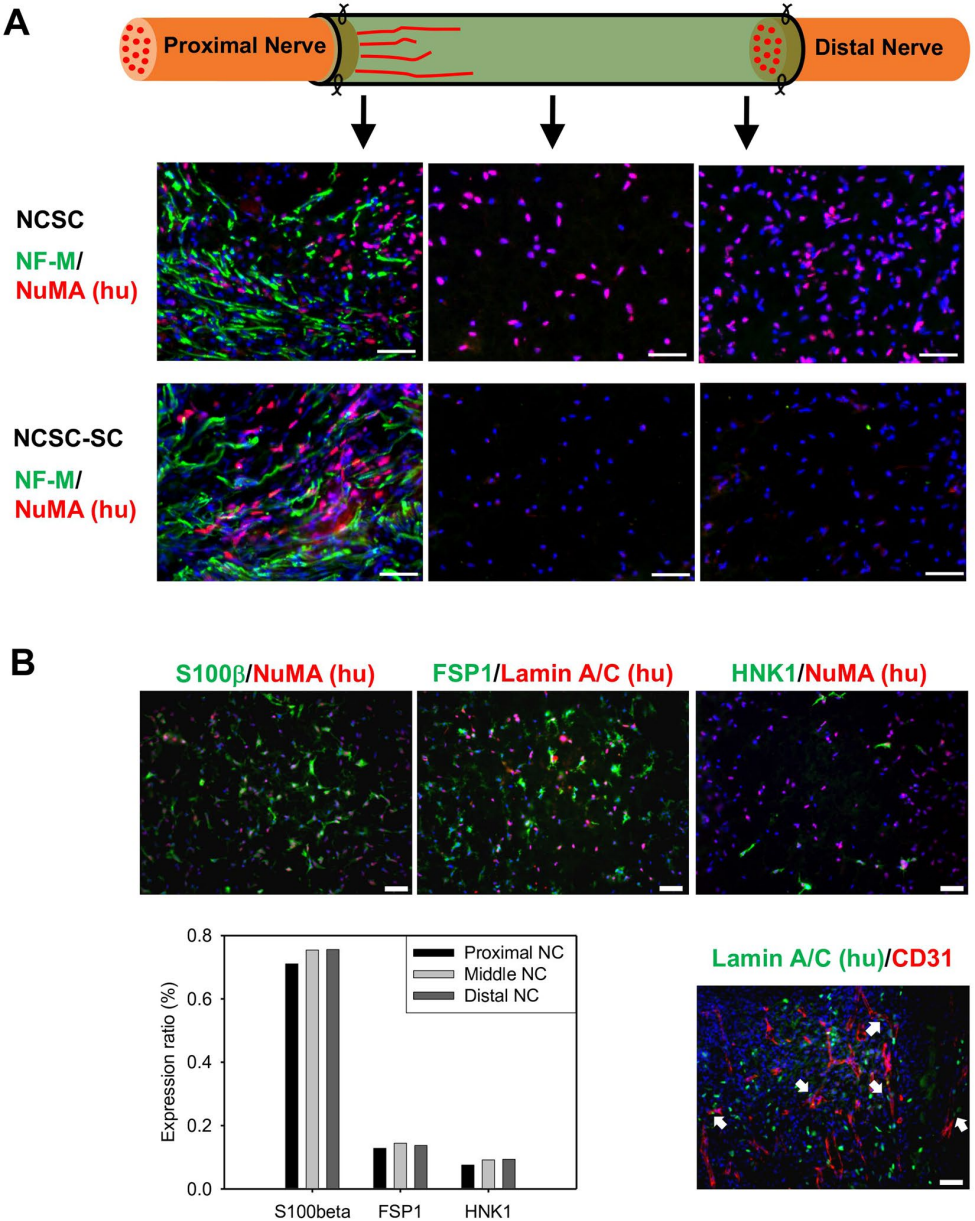
Distribution and behavior of transplanted NCSCs/NCSC-SCs in nerve conduit and NCSC differentiation *in vivo*. (**A**) Immunofluorescence staining of axon marker NF-M and human nuclei antigen NuMA was performed in longitudinally cryosectioned nerve conduits of the NCSC group and the NCSC-SC group at 2-week after surgery. Images were taken at 2–3 mm (left images), 5–6 mm (middle images), and 8–9 mm (right images) of conduit from the proximal end, respectiely. (**B**) Immunofluorescence staining of Schwann cell marker S100β (Green), fibroblast marker FSP1 (Green), NCSC marker HNK-1 (Green), and endothelial marker CD31 (Red) was performed in a longitudinal section of the NCSC group with double staining of human nuclei antigen (NuMA or Lamin A/C). The images in upper row showed marker expression of transplanted NCSC in middle conduit (4–7 mm from proximal end). The bar chart indicated the ratio of S100β/FSP1/HNK-1-positive human cells to total human cells in the examined areas in proximal, middle, and distal conduit. The lower-right image was taken near proximal stump of sciatic nerve in nerve conduit. White arrows pointed the transplanted NCSCs that accompanied the newly growing blood vessels (CD31+). Scale bars are 50μm for all images.

To understand the mechanisms of nerve regeneration, the distribution and differentiation stage of transplanted cells were examined two weeks after surgery. Transplanted NCSCs and NCSC-SCs were identified with immunostaining of human nuclei mitotic apparatus (NuMA). Positive human NuMA staining was observed in both NCSC and NCSC-SC conduits but not in acellular conduits. Transplanted NCSCs were observed throughout the entire length of the conduits (Fig. 4A). However, the transplanted NCSC-SCs were only observed at the proximal end of the nerve conduit (Fig. 4A). Co-staining of axonal marker NF-M and human NuMA showed that both NCSCs and NCSC-SCs integrated with newly growing host axons after 2-week transplantation near the proximal end. Leading edge lengths of the newly growing axons were about 3.5–4 mm from the proximal nerve stump, while no obvious difference in leading edge length was observed between the acellular, NCSC, and NCSC-SC groups at this stage.

The fate of implanted NCSC was determined by immunofluorescence staining for Schwann cell marker S100β, fibroblast marker FSP1, NCSC marker HNK-1, and endothelial marker CD31 with double staining of human NuMA or human Lamin A/C in longitudinal sections of the 2-week NCSC-engrafted conduits (Fig. 4B). Quantification of co-localized lineage specific cell markers and human cell markers showed that a majority of the transplanted NCSCs expressed Schwann cell marker S100β (S100β^+^/hu NuMA^+^, 71.1% in proximal end, 75.4% in middle conduit, 75.6% in distal end). The remaining cells expressed fibroblast marker FSP1 (FSP1^+^/hu Lamin A/C^+^, 12.8% in proximal end, 14.4% in middle conduit, 13.7% in distal end) or NCSC marker HNK-1 (HNK-1^+^/hu NuMA^+^, 7.5% in proximal end, 9.2% in middle conduit, 9.4% in distal end). Some transplanted NCSCs (hu NuMA^+^) were also found to be associated with growing host microvessels (CD31^+^) in the proximal end of the conduit.

### Paracrine Signaling of Transplanted Cells

**Figure 5:**
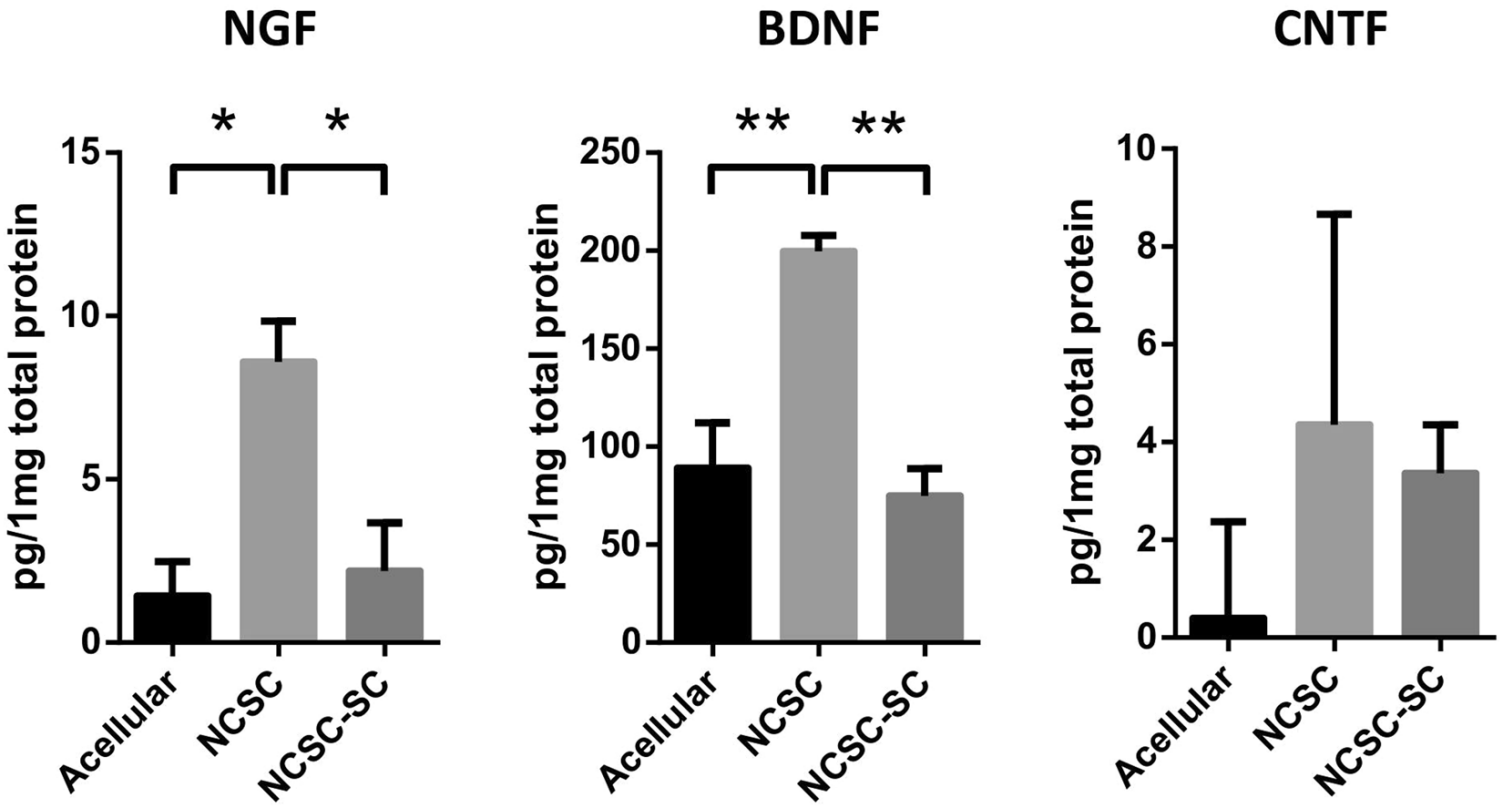
Neurotrophic factors (NFs) Secreted by Transplanted NCSCs/NCSC-SCs. The tissues inside nerve conduits at 2-week after surgery were collected and tested with the expressions of human NGF, BDNF, and CNTF by using sandwich ELISA. Bars represent mean ± standard error of mean. * indicates significant difference from one-way ANOVA (*: p < 0.05; **: P < 0.01; n = 3 for each group).

To determine whether transplanted NCSC and NCSC-SC expressed neurotrophic factors *in vivo*, ELISA was performed in tissue samples collected from within the nerve conduits that were explanted after 2-week transplantation. The expression of human nerve growth factor (NGF), BDNF, and CNTF was quantified by ELISA. As shown in Fig. 5, the amount of NGF per 1 mg total protein was 1.4±1.1 pg, 8.6±1.3 pg, and 2.2±1.5 pg in acellular group, NCSC group, and NCSC-SC group, respectively (Fig. 5). The amount of BDNF per 1 mg total protein was 89.2±23.0 pg, 199.7±8.1 pg, and 75.0±13.9 pg in acellular group, NCSC group, and NCSC-SC group, respectively (Fig. 5). Significant differences in amount of human NGF (p < 0.05) and BDNF (p < 0.01) were observed between acellular group and NCSC group, as well as between NCSC group and NCSC-SC group. The detection of NGF and BDNF in acellular group may due to the non-specific binding of ELISA kit antibodies to endogenous rat BDNF. The amount of human CNTF did not have significant difference in all three groups (Fig. 5).

## Discussion

Functional recovery after segmental peripheral nerve injuries continues to be a challenging clinical problem. Stem cell transplantation has the potential to enhance nerve regeneration, particularly across long injury gaps. However, the differentiation stage of transplanted stem cells could dramatically impact therapeutic safety and efficacy. In this study, we investigated whether the differentiation stage of transplanted iPSC-derived neural lineage cells could impact functional nerve recovery *in vivo*. Since adult Schwann cells has been well studied to help nerve regeneration and NCSCs are a potential source of Schwann cells, we differentiated the human iPSC into NCSCs and subsequently Schwann cells before transplantation to compare the differentiation stage of transplanted cells in their potency to repair injured nerve tissues. The cells were transplanted within a nanofiber nerve conduit and embedded in a composite collagen/hyaluronan hydrogel, both of which have previously been shown to support nerve regeneration *in vivo* ^39,40^.

After 2-week transplantation, we noticed that more human cells were in NCSC-engrafted conduits than that in NCSC-SC-engrafted conduits, indicating a distinct survival advantage for NCSCs over NCSC-SCs after transplantation. In the proximal end of the conduit, the transplanted NCSCs and NCSC-SCs mostly distributed around the newly growing axons, implying their involvement in axon regrowth. In the injured sciatic nerve microenvironment, over 70% of the transplanted NCSCs expressed Schwann cell marker S100β after 2-week differentiation *in vivo*. About 13% of the transplanted human cells in NCSC conduits expressed fibroblast marker FSP1. Fibroblasts are known to play a key role in bridging the injured stumps and inducing the Schwann cells to migrate through the bridge during the early stage of peripheral nerve regeneration^41,42^, which is then followed by axon extension. A smaller amount of the transplanted NCSCs still expressed NCSC marker HNK-1, suggesting that they remained undifferentiated. Interestingly, some transplanted NCSCs were observed to co-localize with newly growing microvessels in the proximal end, thereby suggesting that the transplanted NCSCs may also be involved in angiogenesis within regenerating nerve tissues.

It has been reported that transplantation of rat bone marrow mesenchymal stem cells (BMSCs) in a rat sciatic nerve injury model leads to the increased expression of BDNF and NGF at the injured site^43^. To investigate whether the engrafted iPSC-derived NCSCs and Schwann cells secrete neurotrophic factors (NFs) and participate in paracrine signaling in nerve regeneration, ELISA analyses of human neurotrophic factors NGF, BDNF, and CNTF were performed in the tissues within the implanted nerve conduits. The results showed that BDNF and NGF concentration were higher in the NCSC-engrafted tissues after 2-week transplantation than that in acellular and NCSC-SC groups. The results showed that the NCSC group had significantly higher BDNF and NGF expression than the NCSC-SC group, suggesting that transplanted NCSCs were a better NF-secreting source than NCSC-SCs. On the other hand, both the NCSC and the NCSC-SC groups did not show meaningful expression in human CNTF, implying CNTF is not required for neuromuscular reinnervation after nerve injury^44^. It was noticed that BDNF level in acellular was detectable and high, suggesting that the ELISA kit had cross-reactivity to rat BDNFs. Thus, it is possible that the source of the BDNF expressed in NCSC group could be from the endogenous rat cells which were stimulated by the transplanted human NCSCs.

Further investigation of the effect of neurotrophic factors can provide a more detailed explanation for nerve regeneration. It has been reported that direct delivery of NGF in nerve conduits was performed for nerve regeneration^39^. Their results showed that although NGF can improve neurite outgrowth *in vitro*, nerve conduits incorporated with NGF did not significantly improve peripheral nerve recovery *in vivo*, suggesting that delivery of neurotrophic factors had limited success. An explanation is that the delivery of NGF or limited number of neurotrophic factors does not mimic the effect of NCSC delivery, e.g., cell differentiation and multiple neurotrophic factors secretion. Moreover, we used human M-MSCs as a cell source for nerve regeneration which have been shown to express BDNF and CNTF *in vitro* ^37,38^. The results showed that the M-MSC group did not have better recovery than the acellular group at 1-month, whereas the NCSC group showed significantly improved recovery than the acellular group. In addition, there was no significant difference in muscle mass ratio between the M-MSC group and the acellular group, suggesting that the transplanted M-MSCs with secreted neurotrophic factors may not be sufficient for peripheral nerve regeneration *in vivo*. Taken together, our results suggest that the delivery of neurotrophic factors, either with limited number or undefined release profile, may not be sufficient and that other mechanisms such as NCSC differentiation may be important in addition to paracrine signaling.

BDNF has been demonstrated to possess a wide variety of biological effects on survival, soma size, cholinergic enzymes, and axonal outgrowth of adult motor neurons. In rat spinal root avulsion model, BDNF treatment protects motor neurons from axotomy-induced cell death, and it induces axonal outgrowth of severely damaged motor neurons^45^. BDNF can be secreted by Schwann cells. It plays an essential role in promoting axonal regeneration and re-myelination when Schwann cells were transplanted into nerve injury lesions^46^. In addition, previous studies have shown the essential role of BDNF in peripheral nerve regeneration, blocking BDNF in nerve conduits was performed by applying anti-BDNF antibody, which led to a delay in nerve functional recovery and an impairment in axonal regeneration and myelination^47,48^. There are at least two preferred receptors for mature BDNF, tropomyosin-receptor kinases B (TrkB) and neurotrophin receptor p75 (p75NTR)^49^. p75NTR may serve as a retrograde transport molecule in neurons, promote Schwann cell migration near injury, and/or modulate TrkB activity in those cells that coexpress both p75NTR and TrkB^50,51^. Since human and rat TrkBs exhibit over 90% identity in amino acid sequence^52^, the human BDNF secreted by Schwann cells differentiated from transplanted NCSCs may interact with the host tissues and improve nerve regeneration.

The better integration of NCSCs and the paracrine effects may explain the improved nerve functional recovery in early stages of regeneration (1 month) and more muscle mass at 5 months after transplantation. The amplitude and response latency of the action potential were used to quantify the functional recovery of the regenerated peripheral nerve. The value of CMAP amplitude reflects the number of motor axons activated in the gastrocnemius muscle. The response latency is the time between the onset of the stimulus signal and the beginning of compound muscle action potential. Decreased latency may be related to the enhanced action potential conduction and a better myelination of the regenerated motor axons. The gastrocnemius muscle undergoes significant atrophy in a rat sciatic nerve transection model. Thus, wet gastrocnemius muscle mass measurements may reflect the recovery of gastrocnemius muscle innervation and overall neuromuscular function recovery. The significantly higher CMAP recovery at 1 month and muscle mass recovery at 5 months in NCSC group as compared to acellular group may be explained by rapid nerve regeneration that the NCSC promoted nerve growth across the injury gap at the early stages of nerve regeneration thereby restoring neuromuscular function and reducing atrophy.

To sum up, this study demonstrates that the cell fate and paracrine signaling are related to the stage of NCSC differentiation, which may result in the difference in peripheral nerve regeneration. Transplantation of human iPSC-derived NCSCs has better motor nerve recovery in early stage and long-term muscle recovery than that of mature Schwann cells derived from iPSC-NCSCs for the cell therapy of peripheral nerve injury. These findings have important implications in the selection of appropriate stem cells and their derivatives for stem cell therapies in the regeneration of nerves and other tissues.

## Materials and Methods

### Establishment of Human Integration-free iPS Cell lines

Human adult dermal fibroblasts (HDFa, Life Technologies) were reprogrammed with episomal vectors containing Oct4, Sox2, Klf4, and c-Myc genes by using electroporation method. Electroporation was performed by ALSTEM Bio (Richmond, CA, USA) with their commercial Human iPS Cell Reprogramming Episomal Kit (RF202). In brief, the four episomal vectors were electroporated into HDFa cells which were then reseeded onto gelatin-coated plate to culture on day 0. Puromycin selection was performed from day 1 to day 6 by using Fibroblast Medium supplemented with 0.5 μg/ml of Puromycin. After day 6, the transfected cells were trypsinized on day 6, reseeded onto 6-well plate pre-coated with Matrigel, and then cultured and maintained in mTeSR medium (Stemcell Technologies,05850). About two weeks after transfection, small cell colonies became visible. After three-week culturing, pluripotent stem cell-like colonies were picked up from transfected cells and replated onto Matrigel-coating culture plates. Colonies were expanded for several passage numbers to obtain stable iPS cell lines. Established iPS cell lines were identified with typical pluripotent stem cell morphology such as clear colony border and big nuclei and positive AP staining (Millipore, SCR004). To characterize the integration-free iPS cell lines, immunostaining was performed to examine typical pluripotent stem cell markers OCT4 (Santa Cruz Biotechnology, sc-5279), SOX2 (Millipore, AB5603), SSEA4 (Santa Cruz Biotechnology, sc-21704), TRA-1-60 (Millipore, MAB4360) and TRA-1-81 (Millipore, MAB4381). Nuclei were stained by Hoechst 33342.

### Derivation of neural crest stem cells (NCSCs) from integration-free iPSCs

To obtain human integration-free iPSC-derived NCSCs, the iPSCs were detached and formed embryoid bodies (EBs) in suspension cultures for 6 days using NCSC medium (Life Technologies, A10509–01). After suspension culture, EBs were plated on Matrigel-coating culture plates and kept cultured with NCSC medium for up to 2 weeks. After rosette-like structures were appeared, cells were dissociated into single cells and cultured as monolayer. To obtain homogeneous NCSC populations, magnetic-activated cell sorting (MACS, Miltenyi Biotec) was used to select p75 positive (Miltenyi biotec, 130-097-127) cells. All NCSC lines were further purified by fluorescence-activated cell sorting (FACS, BD Influx) for HNK-1 positive (Sigma, C6680) and SSEA4 (Santa Cruz Biotechnology, sc-21704) negative cells to obtain homogeneous and stable NCSC lines.

### NCSC characterization and Derivation of NCSC-SCs

To characterize iPSC-derived NCSCs, NCSC differentiation towards neuron lineage (peripheral neurons and Schwann cells) and mesenchymal lineage (osteogenic and adipogenic cells) was carried out using the protocol described previously^31^. For NCSC differentiation into peripheral neurons, NCSCs were cultured in knockout DMEM/F12 medium supplemented with N-2 supplement (Invitrogen, 17502–048), Penicillin/Streptomycin, 10 ng/mL brain-derived neurotrophic factor (BDNF, R&D Systems, 248-BD), 10 ng/mL nerve growth factor (NGF, R&D Systems, 256-GF), 10 ng/mL glial cell-derived neurotrophic factor (GDNF, R&D Systems, 212-GD) and 500 μg/mL dibutyryl-cAMP (Sigma-Aldrich, D0260) for 2 weeks. The differentiated cells were immunostained for peripheral neuron markers peripherin and TUJ1.

For osteogenic differentiation, NCSCs were seeded at a low density (10^3^ cells/cm^2^) and grown for 4 weeks in the presence of 10 mM b-glycerol phosphate, 0.1 μM dexamethasone and 200 μM ascorbic acid in DMEM medium supplemented with 10% FBS and Penicillin/Streptomycin. Then cells were fixed in 4% paraformaldehyde and stained with Alizarin Red (A5533, Sigma) for calcified matrix. For adipogenic differentiation, confluent NCSCs were treated with 10 μg/mL insulin, 1 μM dexamethasone and 0.5 mM isobutylmethylxanthine in DMEM medium supplemented with 10% FBS and Penicillin/Streptomycin for 3 weeks, and cells were stained with oil red (O0625, Sigma) for lipid and fat deposited by the cells.

To obtain NCSC-derived Schwann cells, iPSC-NCSCs were cultured in knockout DMEM/F-12 medium supplemented with N-2 supplement, Penicillin/Streptomycin, 10ng/mL ciliary neurotrophic factor (CNTF, R&D Systems, 257-NT), 10ng/mL basic Fibroblast Growth Factor (bFGF, Peprotech, 100–18B), 500 μg/mL dibutyryl-cAMP (dbcAMP, Sigma, D0260) and 20ng/mL neuregulin (NRG, R&D Systems, 377-HB). The cells were immunostained periodically with Schwann cell markers GFAP and S100β to monitor the differentiation stage. Schwann cells obtained after 3-week differentiation were used for *in vivo* transplantation.

### Nerve conduit fabrication

Electrospinning technique was used to produce nanofibrous nerve conduits. Nonwoven aligned nanofibrous nerve conduits composed of poly(L-lactide-co-caprolactone) (70:30, Purac Biomaterials), poly(propylene glycol) (Acros Organics) and sodium acetate (Sigma) were fabricated by using a customized electrospinning process. To make tubular scaffolds with aligned nanofibers in the longitudinal direction on luminal surface, a rotating mandrel assembly with two electrically conductive ends and a central non-conductive section was used. The jet stream of polymer solution from the spinneret whipped between the two conductive ends, resulting in longitudinally aligned nanofibers forming a tubular scaffold on the non-conductive portion of the mandrel. To enhance the mechanical strength of the scaffolds, outer layers of random nanofibers were deposited on this layer of longitudinally aligned fibers^36^.

### Hydrogel matrix fabrication

HA crosslinker MMP peptides (GCREG-PQGIWGQ-ERCG) were purchased from GenScript. Sodium Hyaluronate (60 kDa) was purchased from Lifecore Biomedical (HA60k-1), rat tail collagen I from Corning (354249), divinyl sulfone from Sigma-Aldrich (V3700), Ellman’s reagent from Fisher Scientific (PI22582).

Vinyl sulfone modified hyaluronic acid (HA-VS) was prepared using one-step synthesis^53^. Sodium hyaluronate (240 mg, 0.2 mmol, 60 kD) was reacted with divinyl sulfone (602 ul, 1.3 mol) in 0.1 N NaOH solution for 26 minutes at room temperature. The reaction was stopped by 600ul 4 N HCl, and the product was purified through dialysis (10kD MWCO) against 0.15 M NaCl solution overnight and then against DI water overnight. The purified intermediate (HA-VS) was lyophilized and stored at 4 °C until used. The vinyl sulfone (VS) content at modified hyaluronic acid polymer was determined by quantitating sulfhydryl groups using Ellman’s Reagent and a cysteine standard. Based on the assay, 20.1% of the hydroxyl groups were modified with vinyl sulfone. The HA-VS polymers can then be crosslinked with bis-cysteine containing peptide crosslinkers to form hydrogel.

### Tissue engineered conduits preparation

The cells were embedded in a collagen-hyaluronic acid (HA) hydrogel carrier and injected into the lumen on the PLCL conduit. The PLCL conduits and modified HA powder were sterilized by ethylene oxide gas sterilization before use.

HA-VS was dissolved in 0.3 M Triethanolamine (TEA) buffer and pre-mixed with collagen I solution, 10X PBS, and 1 N NaOH. NCSCs were detached and re-suspended in the serum-free NCSC maintenance medium in the concentration of 4 × 10^4^ cells/μl. The cell suspension was then mixed with the HA and collagen solution. Then lyophilized MMP peptides were dissolved in 0.3 M TEA buffer and immediately mixed with the hydrogel-cell mixture. The final concentration of HA was 4 mg/ml and collagen I was 4 mg/ml, the final pH value of the hydrogel was 7, and the crosslinking density of HA-VS was 0.7 (moles of -SH from the crosslinkers over moles of -VSs from the HA-VSs). The hydrogel precursor solution was injected into the nanofibrous nerve conduits (25ul hydrogel with 0.5million cells per conduit). The tissue-engineered constructs were kept in the incubator (37 °C) for 1 hour for gelation, and cell culture media were then added to cover the constructs. The culture was maintained in the incubator overnight before surgery.

### *In vivo* transplantation of stem cells and nerve conduits

In this study xenogenic transplantation was performed since no allogenic model is available for human cells due to ethical reasons. All experimental procedures with animals were approved by the ACUC committee at UC Berkeley and were carried out according to the institutional guidelines. Adult female athymic nude rats (Charles River) weighing 200–250 g were used in all experiments, and 12 animals were used in each group. Three experimental groups were included: (1) conduits filled with collagen-HA hydrogel without cells (control group), (2) conduits seeded with NCSCs (experimental NCSC group), and (3) conduits seeded with NCSC-SCs (experimental NCSC-SC group). For nerve conduit implantation, an incision was made over the skin of the hip joint with a sterile scalpel. Under a surgical microscope, the sciatic nerve was severed with a scalpel at two spots to make a 1-cm gap. Then a nerve conduit (11 mm in length, 1.5 mm in diameter) was inserted between the two nerve stumps and sutured with 8–0 nylon monofilament sutures. The overlying muscle layers and skin were sutured with 4–0 absorbable sutures to close the surgery site. After 2 weeks, 6 animals of each group were euthanized and the conduits were harvested. Three conduits per group were snap-frozen with liquid nitrogen for ELISA test, the other three conduits were fixed in 4% paraformaldehyde for immunohistological analysis. After 1-month and 5-month transplantation, nerve regeneration was assessed by electrophysiology, and muscle recovery was assessed by wet gastrocnemius muscle weight.

### Electrophysiology

Electrophysiology testing was performed following the previous methods^54^. In brief, the rat sciatic nerve was re-exposed and electrical stimuli (single-pulse shocks, 1 mA, 0.1ms) were applied to the native sciatic nerve trunk at the point 5 mm proximal to the graft suturing point. CMAPs were recorded on the gastrocnemius belly from 1 V to 12 V or until a supramaximal CMAP was reached. Normal CMAPs from the un-operated contralateral side of sciatic nerve were also recorded for comparison. Grass Tech S88X Stimulator (Astro-Med Inc.) was used for the test and PolyVIWE16 data acquisition software (Astro-Med, Inc.) was used for recording. Recovery rate is the ratio of injured hindlimb’s CMAP to contralateral normal hindlimb’s CMAP of a rat.

### Histological analysis and immunostaining

The nerve conduits were harvested and fixed in 4% paraformaldehyde at 4 °C for 2 hours. After been washed with PBS, tissues were cryoprotected with 30% sucrose in PBS at 4 °C overnight, and were then embedded in optimum cutting temperature (OCT) compound and were frozen in −80 °C. The frozen samples were cryosectioned longitudinally and transversely in −20 °C in the thickness of 10 μm. The slices were placed onto Superfrost plus slides and stored in −20 °C.

Immunostaining was performed for histological analysis. Slices were permeabilized with 0.5% Triton X100 in PBS for 30 min, blocked with 4% normal goat serum in PBS for 1 h, and then incubated overnight at 4 °C with primary antibodies. Slides were then washed with PBS and incubated with secondary antibodies for 1 h at room temperature. After further PBS washing, coverslips were mounted and viewed with a fluorescent microscope (Zeiss). The primary antibodies used for immunohistochemistry in this study were: NFM (ab7794, Abcam), S100β (ab4066, Abcam), NuMA (ab84680, Abcam), FSP1 (ab27957, Abcam), HNK-1 (C6680, Sigma), human lamin A/C (MAB1281, Millipore), CD31 (ab28364, Abcam). Besides, Abcam goat anti- mouse and goat anti-rabbit secondary antibodies were used.

### Enzyme-linked immunosorbent assay (ELISA)

Solid phase sandwich ELISA was performed to detect neurotrophic factors in nerve conduits. The snap-frozen 2-week conduits were thawed on ice. The tissue inside conduit was then collected, soaked in 500 μl tissue lysis buffer (786–180, Mammalian Cell PE LB, G-Biosciences) supplemented with protease inhibitors (1 mM PMSF, 1 mM Na_3_VO_4_, and 10 μg/ml Leupeptin), and then homogenized with GentleMACS dissociator. The tissue lysate was centrifuged under 14000 × g for 15 min at 4 °C to remove debris, and the supernatant was collected for ELISA test. Human BDNF Quantikine ELISA kit (DBD00, R&D Systems), human CNTF Quantikine ELISA kit (DNT00, R&D Systems), and human beta-NGF DuoSet ELISA kit (DY256–05, R&D Systems) were used for the tests following manufacturer’s instruction. The amount of growth factor was normalized by total protein concentration of tissue lysate for comparison. Total protein concentration of tissue lysate was determined by DC™ Protein Assay (BioRad) prior to ELISA.

### Statistical analysis

The data were reported as mean ± S.E.M., unless otherwise described. Comparisons among values for groups greater than two were performed by one-way analysis of variance (ANOVA), and differences between groups were then determined using a Tukey’s *post hoc* test. For two groups analysis, a two-tailed, unpaired Student’s *t*-test was used for the analysis of differences. For all experiments, a value of *p* < 0.05 was considered statistically significant. GraphPad Prism software was used for all statistical analyses.

## Electronic supplementary material

Supplementary Information
