# Supplementary material for: The Differentiation Stage of Transplanted Stem Cells Modulates Nerve Regeneration
Source: Sci Rep. 2017 Dec 12;7:17401. doi: 10.1038/s41598-017-17043-4 (PMC5727226; doi:10.1038/s41598-017-17043-4)
Supplement: Supplementary file 1 — Supplementary Information [file 41598_2017_17043_MOESM1_ESM.pdf]

The Differentiation Stage of Transplanted Stem Cells Modulates Nerve Regeneration

Ching-Wen Huang[1,†], Wen-Chin Huang[1,2,†], Xuefeng Qiu[1,3,†], Flavia Fernandes Ferreira da Silva[4], Aijun Wang[5], Shyam Patel[1], Leon J. Nesti[6,7,8], Mu-Ming Poo[9], Song Li[1,10,11,*]

[1]Department of Bioengineering, University of California, Berkeley, California 94720, USA
[2]UC Berkeley-UCSF Graduate Program in Bioengineering, Berkeley, California 94720, USA
[3]Department of Cardiovascular Surgery, Union Hospital, Tongji Medical School, Huazhong University of Science and Technology, Wuhan 430022, China
[4]Instituto de Macromoléculas Professora Eloisa Mano, Universidade Federal do Rio de Janeiro, Rio de Janeiro, Brazil
[5]Department of Surgery, University of California, Davis, School of Medicine, Sacramento, California 95817, USA
[6]Department of Surgery, Uniformed Services University of the Health Sciences, Bethesda, Maryland 20814, USA
[7]Clinical and Experimental Orthopaedics, National Institute of Arthritis and Musculoskeletal and Skin Diseases, National Institutes of Health, Bethesda, Maryland 20892, USA
[8]Department of Orthopaedic Surgery, Walter Reed National Military Medical Center, Bethesda, Maryland 20889, USA
[9]Department of Molecular and Cell Biology, University of California, Berkeley, California 94720, USA
[10]Department of Bioengineering, University of California, Los Angeles, California 90095, USA
[11]Department of Medicine, University of California, Los Angeles, California 90095, USA

[†] These authors contributed equally to this work.

[*] Correspondence to: Dr. Song Li, songli@ucla.edu

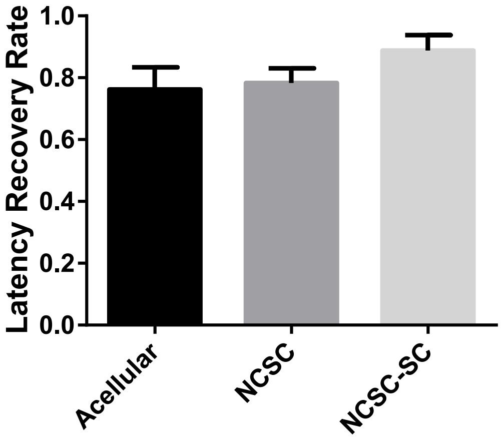

Figure S1. *In vivo* evaluation of latency recovery at 1-month after surgery in experimental groups with or without cell transplantation into nerve conduits.

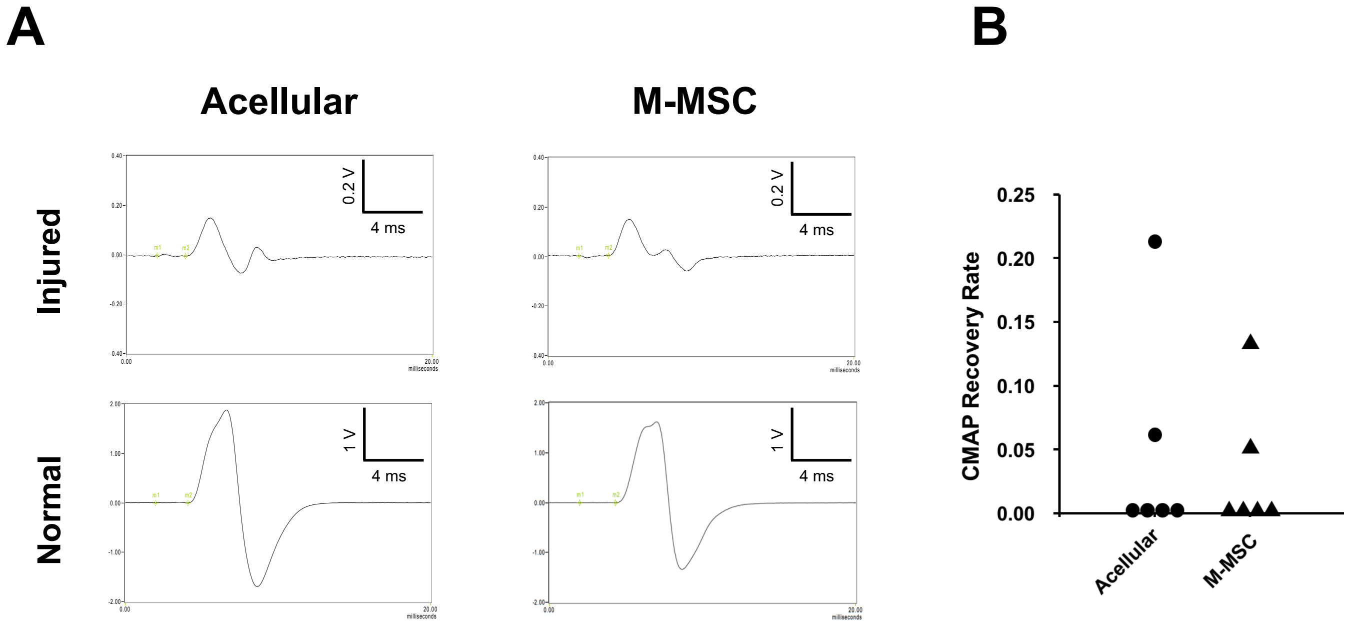

Figure S2. *In vivo* evaluation of functional recovery with or without M-MSC transplantation into nerve conduits. (A) Compound muscle action potential (CMAP) was measured *in vivo* at 1-month after surgery. Representative CMAP curves of the acellular and M-MSC groups are shown. (B) CMAP recovery rate of individual rats (n=6 for acellular group and M-MSC group)

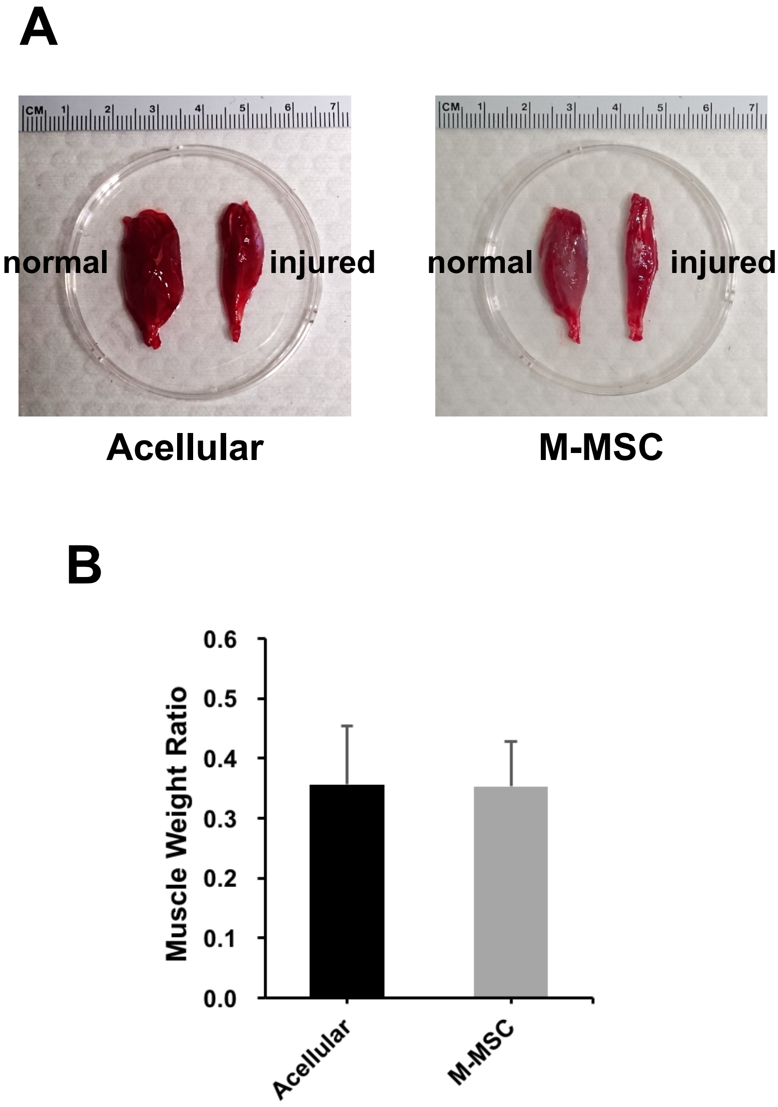

Figure S3. *In vivo* evaluation of functional recovery with or without M-MSC transplantation into nerve conduits. (A) Representative images of gastrocnemius muscle are shown for the acellular group and the M-MSC group at 2-month after surgery. (B) Bars represent mean ± standard error of mean. (n=6 for acellular group and M-MSC group).
